# Systematic review of accuracy of reporting of Congo red-stained amyloid in 2010–2020 compared with earlier

**DOI:** 10.1080/07853890.2022.2123558

**Published:** 2022-09-18

**Authors:** Alexander J. Howie, Mared P. Owen-Casey

**Affiliations:** a Department of Pathology, University College London, London, UK; b Betsi Cadwaladr University Health Board, Wales, UK

**Keywords:** Systematic review, amyloid, anomalous colours, Congo red, polarisation microscopy

## Abstract

**Background:**

Almost always, Congo red-stained amyloid between polariser and analyser is said to show “green birefringence” or “apple-green birefringence”. In 2010, we found that not all published images showed green, and not all that did showed only green. This systematic review of more recent papers was to find if there had been any improvement in the accuracy of reporting.

**Materials and Methods:**

MEDLINE was searched on 15 March 2021 for papers published between 2010 and 2020 inclusive mentioning amyloid and Congo red. These were examined for descriptions of colours, which were compared with images. Papers were searched for mentions of anomalous colours, errors in physical optics, and misquotation of references about polarisation.

**Results:**

In 374 papers, there were 444 descriptions of colours, with 511 images in 257 papers. The commonest descriptions were apple-green, 249**/**444 (56%), and green, 105**/**444 (24%). The description agreed with colours seen in 116/511 images (23%) (previously 64/191, 34%). Green was seen in 342**/**511 images (67%) (previously 159/191, 83%), but not in 169**/**511 (33%), although each image was reported to show green. Green alone was seen in 103**/**511 images (20%) (previously 59/191, 31%), and was combined with at least one other colour in 239**/**511 (47%). Ten papers included the term anomalous. Eight papers incorrectly said that there was green dichroism, three incorrectly used the term green metachromasia, and two incorrectly mentioned green fluorescence. Twenty-seven papers misquoted references.

**Conclusions:**

There is widespread and increasing inaccuracy of reporting of colours seen in Congo red-stained amyloid. People persist in saying “green birefringence” or “apple-green birefringence”, even when no green is seen, or there are also other colours. Few appear to appreciate that the other colours are genuine, respectable, and helpful, the physical optical principles that explain the colours are now understood, and the best expression to use is anomalous colours.

**KEY MESSAGE**
“Green birefringence” and “apple-green birefringence” are inappropriate terms to describe the findings in amyloid stained with Congo red and examined between crossed polariser and analyser, because green is not always seen, and even when it is, other colours are commonly seen as well. The proportions of colour images showing any green and green alone, and the proportion of descriptions that agreed with illustrated colours, significantly decreased in 2010–2020 compared with earlier. The most appropriate and scientific description of the findings is anomalous colours.

## Introduction

Most people working on amyloid say that it shows “green birefringence” or “apple-green birefringence” when stained with Congo red and examined between crossed polariser and analyser [1]. In this text, green includes apple-green, unless these are specifically differentiated. Green birefringence is commonly thought to be essential for the diagnosis of amyloid. This is because in the early days of interest in the optical properties of Congo red-stained amyloid there was a mistaken and dogmatic insistence that only green should be seen to make the diagnosis. There was also an insistence that no other colour should be seen, and as a result, usually no other colour was mentioned. These ideas have persisted [1–5].

Unfortunately, these ideas are wrong, as can be easily confirmed by inspection of published images of Congo red-stained amyloid. If green is indeed necessary for the diagnosis, every relevant illustration should show green. Because green is almost always the only colour mentioned, with the implication that no other colour is seen, most illustrations should only show green. In 2010 we published a study of 160 papers on Congo red-stained amyloid containing 191 colour images, which suggested widespread inaccurate and unscientific reporting, because, as examples, not all images showed green, few showed green alone, and two thirds had a discrepancy between colours claimed to be seen in images and what was actually illustrated [1].

The simplest and most scientifically accurate way to describe what is seen is to say that there are anomalous colours, which means colours different from the colour of Congo red in ordinary illumination, not related to Newton’s scale of interference colours [6]. The physical optical principles that explain the colours, and how they change, for example as the polariser and analyser are rotated, have been fully described in papers which do not require readers either to have a specialised knowledge of physics or to refer to other papers or texts [1–5].

Because some time had passed since the 2010 study, which included papers published in 2009 and earlier, the objective of this systematic review was to see whether there had been any change in reporting of findings in more recent papers, and in particular, whether the accuracy of reporting of what was seen had improved. The review follows the updated guideline of the Preferred Reporting Items for Systematic Reviews and Meta-Analyses (PRISMA) statement (2020) [7].

## Materials and methods

**Figure 1.**
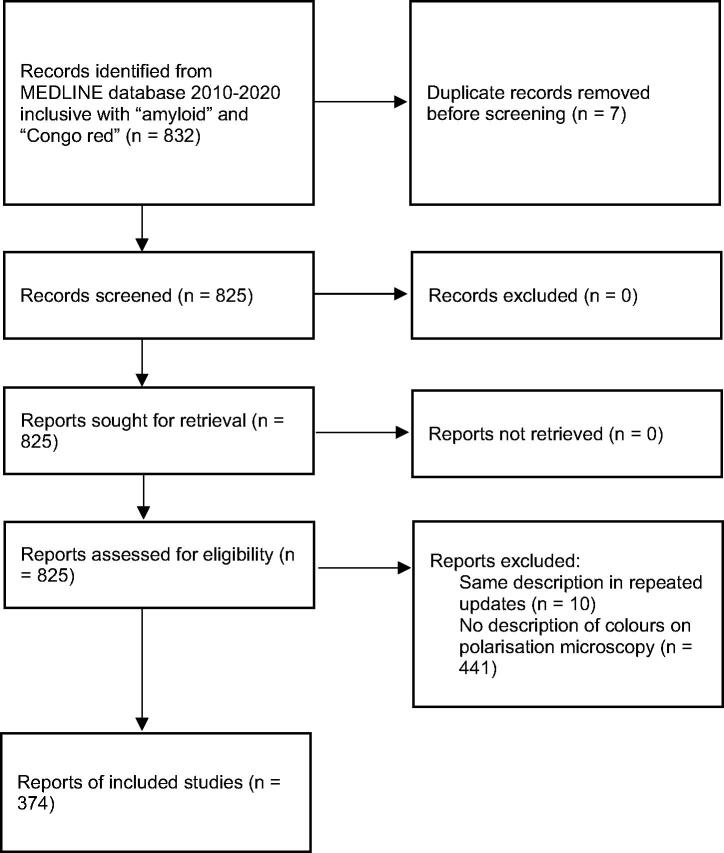
PRISMA 2020 flow diagram for systematic review of papers published in 2010–2020 inclusive reporting colours seen on polarisation microscopy of Congo red-stained amyloid.

MEDLINE was accessed on 15 March 2021 and searched for papers which included the words amyloid and Congo red, published between 2010 and 2020 inclusive. The flow diagram (Figure 1), as required by PRISMA (2020), shows how the papers selected for inclusion in the systematic review were identified. Those with a description of colours seen on polarisation microscopy were identified. In descriptions of images, apple-green and green were considered synonyms. This group was then searched for papers with at least one relevant colour image. PDF images**, **rather than high resolution web images, which were not often available, were inspected to determine whether (1) there was agreement between the colours described, and what was seen; (2) green could be seen, and if so, whether it was on its own; and (3) green was said to be in the image, but could not be seen. Two observers separately examined each image and then compared notes. Any differences were reconciled by discussion, and were generally settled by agreement that if either observer thought they saw any green, the image was accepted to show green. As before [1], in cases of doubt, the colour was called green. The differences between the current and previous findings [1] were compared using the χ^2^ test.

The text of papers with a description of colours was examined to see whether (1) anomalous colours were mentioned; (2) there were mistakes in the description of the optical properties, apart from discrepancies in the description of colours; and (3) the references used to support any statement about the optical properties were quoted correctly.

## Results

### Study selection

In the 11 years 2010 to 2020 inclusive, 832 papers were identified that included the words amyloid and Congo red. After exclusion of duplicates, the text of 825 papers was searched to see whether there was a description of at least one colour seen in Congo red-stained amyloid between crossed polariser and analyser, which was usually just said to be on polarisation or similar expressions. Ten papers were excluded which were repeats of updates with the same description each time, and 441 publications that did not mention a colour were discarded. This gave 374 papers that were included in this review. These were further searched to see if they contained at least one colour image of Congo red-stained amyloid between crossed polariser and analyser.

In the 374 papers, there was a total of 444 descriptions of colours, because several papers gave more than one description. These included 257 papers with at least one relevant colour image, with a total of 511 images. There were 117 papers that did not have colour images. Supplement 1 lists the 257 papers with images and their descriptions, plus the colours identified in images by the current authors. Supplement 2 lists the 117 papers with descriptions but no images. The supplements also note if papers mentioned anomalous colours, or made mistakes about the physical optics, or misquoted references about polarisation microscopy.

### Descriptions and illustrations of colours

The commonest colour mentioned was apple-green, in 249 of 444 descriptions (56%), followed by green, in 105**/**444 (24%). Apple-green was described significantly more than green compared with previously [1], when there were 71**/**177 (40%) descriptions of apple-green and 85**/**177 (48%) descriptions of green (χ^2^ = 35.8, degrees of freedom (d.f.) = 2, *p* < 0.001).

Taking green and apple-green as synonymous in descriptions, the description agreed with the colours seen in 116/511 images (23%) (previously 64/191, 34%), with a discrepancy in 395**/**511 (77%). The difference between the studies is significant (χ^2^ = 8.5, d.f. = 1, *p* < 0.005). Most discrepancies were between a description of just green or apple-green and an appearance of at least two colours in the image, not necessarily including green, which applied to 230/395 discrepancies (58% of discrepancies). Other discrepancies included a description of just green or apple-green but another single colour in the image, description of a colour or colours other than just green or apple-green but illustration of a different colour or different colours, and images that were too poor to allow identification of any colour.

The observers accepted that 342 images (67% of 511) showed any green (previously 159/191, 83%), while 169**/**511 (33%) did not, although each of these images was reported to show green. The difference between the studies is significant (χ^2^ = 18.1, d.f. = 1, *p* < 0.001). Green alone was seen in 103**/**511 images (20%) (previously 59/191, 31%), and was combined with at least one other colour in 239**/**511 (47%), in which the commonest combination was green and yellow. The difference between the studies is significant (χ^2^ = 20.2, d.f. = 2, *p* < 0.001). Several colours other than green and yellow were seen in images, including blue, orange, red, and white, on their own or in a variety of combinations.

### Anomalous colours, optical mistakes and misquoted references

Ten papers included the term anomalous. This was usually applied correctly as a description of a colour or more than one colour, such as, “Amyloid deposits are Congo red positive (orangiophilic) and produce apple green birefringence or other anomalous colors under polarized light” [8]. Some authors, though, were too restrictive in descriptions, such as, “When Congo red binds to amyloid it becomes birefringent due to its orientation and optical properties, visualized as anomalous yellow-green or orange colors under crossed-polarizers” [9]. This is too restrictive because there is a wider range of possible anomalous colours and combinations of them [1–5].

The birefringence of Congo red-stained amyloid is shown by the appearance of brightness against a dark background when the specimen is examined between crossed analyser and polariser [1–5]. Eight papers incorrectly said that there was green dichroism. Dichroism is shown with either an analyser or a polariser, but not both, and means that a material absorbs some wavelengths of light polarised in one direction of orientation of the material, and so appears a particular colour, red in the case of Congo red-stained amyloid, but does not absorb light polarised at right angles, and so the material appears in theory colourless, but in practice a lighter shade of the same colour, still red in this case [3]. Three papers used the term green metachromasia when talking about polarised light, but metachromasia means that there is a change of colour when a dye is examined in ordinary, unpolarised light [5]. Two papers used the term green fluorescence in polarised light, but fluorescence means emission of longer wavelengths, usually in the visible spectrum, than the illuminating wavelengths, usually ultraviolet radiation.

There were 27 papers that misquoted references about polarisation microscopy. Some incorrectly attributed to earlier papers statements that had been passed uncritically from paper to paper [5]. For example, one paper said, “In the 1920s Bennhold introduced polarized microscopy and showed typical apple-green birefringence” [10]. In fact, Bennhold, who discovered that Congo red stained amyloid, never used polarisation and so could not show the birefringence or birefringent colours of Congo red-stained amyloid [5]. Other authors supported claims of colours by wrongly quoting papers. For example, one paper which said that there was “characteristic apple-green birefringence when Congo red-stained amyloids are examined between crossed polarizer and analyzer” [11] had misunderstood the reference, which in fact showed that there was no such thing as “characteristic apple-green birefringence” [2].

## Discussion

We have shown not only that there is widespread inaccuracy of reporting of what is seen in Congo red-stained amyloid on polarisation microscopy, but also that the inaccuracy is significantly increasing. Compared with our findings in the years before 2010 [1], we found that in 2010 to 2020 inclusive, discrepancies between what was illustrated and what was claimed to be seen increased from 66% to 77% of figures, the proportion of figures showing no green increased from 17% to 33%, and the proportion showing green alone decreased from 31% to 20%. This inaccurate and unscientific reporting reflects badly on the objectivity of observers, who rather than relying on their own experience and questioning received opinions, which is what clinical scientists such as physicians and pathologists are expected to do, report what long-standing but erroneous tradition has conditioned them to see and to report.

With so many papers in the systematic review, if any had been missed, say, that other databases would have identified, or if any had been wrongly categorised, the proportions of discrepancies are unlikely to be misleading to an important amount. Even though interpretation of colours is subjective, most discrepancies were between descriptions of a single colour in an image, almost always green or apple-green, and two or more colours included in the image, which observers had no doubt and agreed were multiple. There was consistency between the current study and the previous one [1], because the observers were the same. As in that study, images were given the benefit of doubt if there was uncertainty if they showed green, and so, if anything, the proportions showing green alone, or any green, were probably overestimated rather than underestimated. No authors said that the published colours had misrepresented what they had seen, or said that high resolution web images were more representative than PDF images, and so their descriptions of colours were taken at face value.

We have also shown that most people persisted in saying “green birefringence”, or more commonly, “apple-green birefringence”, even when there was no green in an image, or there were other colours that were ignored. The Nomenclature Committee of the International Society of Amyloidosis recognised in 2020 that “in reality, red, green and yellow are commonly seen” and “green may be very weak and difficult to see”, and so recommended that “the findings should be described in detail in order to avoid a statement that is not fully correct” [12]. This recommendation is admirable, but is unlikely to be widely followed, if only because authors prefer to give a short description, rather than a detailed and accurate but long account of the colours they actually see, which are almost always multiple and change as the optical arrangements alter. In fact, there is a scientifically accurate term that is shorter than either “green birefringence” or “apple-green birefringence”, namely, anomalous colours, which does not require each colour to be specified [1–6]. These colours are genuine, respectable, and helpful, and should not be ignored, despite the widespread, traditional obsession with “green birefringence” or “apple-green birefringence”.

Anomalous colours have started to be mentioned in papers, although there is evidence that the physical optical principles of Congo red-stained amyloid are still misunderstood by many authors. This is shown by the misuse of the technical expressions, dichroism, metachromasia, and fluorescence, and by the misquotation of either historical references, or references that explain the physical optical principles underlying the colours [1–5]. “Green birefringence” became the supposedly best and obligatory expression in the 1950s. This was incorrect, because the physical optical principles were not fully understood by those who introduced and insisted on the term, as shown by these examples of errors: the idea that green was specific for Congo red-stained amyloid; an unrealistic and unnecessary insistence that only a microscope specifically designed for polarisation microscopy should be used; mistaken explanations of the findings; and imposition of a rigid rule that it was a mistake to see colours other than green [5]. “Apple-green birefringence” was introduced in the 1970s, although the specific type of green apple has never been defined. Few authors had the knowledge or confidence to challenge the accepted dogma of “green birefringence” or “apple-green birefringence”, even when they saw other colours, or even saw no green at all [1, 5].

## Conclusions

There is still a widespread insistence that Congo red-stained amyloid shows “green birefringence” or, more usually, “apple-green birefringence”, when examined between polariser and analyser, even though green is not always seen, and even when it is, is not always on its own. Other colours are frequently overlooked. Observers have been trained that green is essential for the diagnosis of amyloid. Accordingly, they expect to see and report it, even when it is not there. Why authors persist in this is an interesting example of force of habit, or unquestioning acceptance of orthodoxy, in the face of everyday evidence to the contrary. Physical optical principles can explain the range of colours seen, which should be called anomalous colours.

## Supplementary Material

Supplemental Material

Supplemental Material

## Data Availability

There are two supplements. Supplement 1 lists the 257 papers with figures, and Supplement 2 lists the 117 papers without figures. Both supplements mention how colours were described, the reference, and, if appropriate, a note of whether the word anomalous was included, or whether the optical properties were incorrectly described as dichroism or metachromasia or fluorescence, or whether a paper was misquoted, with the reference of the misquoted paper. In Supplement 1, the colours seen in images by the authors of this review are given, plus whether these agree with the description or there is a discrepancy, plus a note if no green was seen. Further details can be supplied on receipt of any reasonable request to the corresponding author.

## PRISMA guidelines for systematic review

The authors confirm that they followed the PRISMA guidelines (https://www.equator-network.org/reporting-guidelines/prisma/).
