# Supplementary material for: Systematic review of accuracy of reporting of Congo red-stained amyloid in 2010–2020 compared with earlier
Source: Ann Med. 2022 Sep 18;54(1):2511–6. doi: 10.1080/07853890.2022.2123558 (PMC9518257; doi:10.1080/07853890.2022.2123558)
Supplement: Supplemental Material [file IANN_A_2123558_SM2178.docx]

**Systematic review of accuracy of reporting of Congo red-stained amyloid in 2010-2020 compared with earlier**

**Supplement 2: papers with descriptions but no figures**

Alexander J Howie and Mared P Owen-Casey

Each entry is in this order: Number in this Supplement; name of first author; if relevant, WRONG EXPRESSION (dichroism or metachromasia), or MISQUOTE of a reference, or ANOMALOUS if mentioned; description of colour(s) in text; reference; [if relevant, misquoted reference]

**n = 117**

1 Agarwal apple green birefringence AutopCaseRep 2020; 10: e2019133.

2 Alkhawam green birefringence; orange-green birefringence ActaCardiol 2017; 72: 380-389.

3 Amin apple-green birefringence KobeJMedSci 2014; 60: E5-E11.

4 Azarfar apple-green birefringence ModRheumCaseRep 2021; 5: 117-122.

5 Balwani apple green birefringence JNeuropharm 2015; 4: 69-71.

6 Basu MISQUOTE apple green birefringence IntJBiolMacro 2018; 107: 2643-2649. [Ladewig P. Double-refringence of the amyloid-congo-red-complex in histological sections. Nature 1945; 156: 81-82.]

7 Baumgart MISQUOTE characteristic, typical green-yellow-orange birefringence VirchArch 2018; 473: 627-637. [Howie AJ, Brewer DB. Optical properties of amyloid stained by Congo red: History and mechanisms. Micron 2009; 40: 285-301.]

8 Bernabei apple-green birefringence Medicine 2020; 99: e18978.

9 Bernardez green birefringence JCutPath 2015; 42: 496-502.

10 Caruana apple-green birefringence BMJCaseRep 2019; 12: 6.

11 Castellani weak apple green birefringence VirchArch 2017; 470: 455-463.

12 Chee characteristic apple-green birefringence ClinLymphMyelLeuk 2010; 10: 177-180.

13 Comenzo apple-green birefringence Blood 2014; 123: 1436-1437.

14 Cornejo apple-green birefringence AmJDermpath 2015; 37: e129-e133.

15 Dapson WRONG and ANOMALOUS apple green and anomalous colors; the birefringence is colored (dichroic) BiotechHistoch 2018; 93: 543-556.

16 Das typical, characteristic apple‑green birefringence IndJOphthal 2019; 67: 2078-2080.

17 Dasari apple green–orange birefringence KidInt 2016; 90: 658-664.

18 de Almeida apple green birefringence, red-green birefringence Lung 2015; 193: 875-883.

19 de Mello apple-green birefringence SaoPaulMedJ 2011; 129: 176-180.

20 Demiraslan apple-green birefringence SAfrJHIVMed 2017; 18: 735.

21 Diaz green birefringence; typical apple-green birefringence ArabJGastr 2018; 19: 96-99.

22 Dutta green birefringence EndokrylPol 2015; 66: 555-558.

23 Elhensheri green birefringence JZooWildMed 2012; 43: 181-185.

24 Fernandez de Larrea typical apple-green birefringence Blood 2015; 125: 2239-2244.

25 Fernandez-Flores ANOMALOUS yellow to green, apple-green birefringence, anomalous colours, green birefringence BiotechHistoch 2011; 86: 293-301.

26 Freudenthaler characteristic, typical green-yellow-orange birefringence VirchArch 2016; 468: 569-577.

27 Gallegos apple-green birefringence CurrCardiolRep 2020; 22: 40.

28 Ganapathy apple-green birefringence Cureus 2019; 11: e3828.

29 George apple-green birefringence WorldNeurosurg 2020; 142: e325-e330.

30 Gertz apple-green birefringence AmJHem 2020; 95: 848-860.

31 Gillmore classical apple green birefringence, pathognomonic green birefringence BrJHaem 2015; 168: 207-218.

32 Girych characteristic green–yellow birefringence MethApplFluor 2016; 4: 34010.

33 Gonzalez apple-green birefringence BMJCaseRep 2018; 2018/10/23.

34 Gonzalo-Garijo apple-green birefringence JClinImm 2014; 34: 119-122.

35 Gowda characteristic apple-green birefringence JBiolmolNMR 2017; 69: 207-213.

36 Hamley apple-green birefringence JPhysChemB 2010; 114: 8244-8254.

37 Herrera birefringence (ideally green but yellow is acceptable) UltrastrPath 2020; 1-17.

38 Hill apple-green birefringence FrontAgNeurosci 2015; 7: 9.

39 Hirano apple-green birefringence BMCPulmMed 2018; 18: 77.

40 Ikezawa green birefringence; yellowish-green birefringence IntJSurgPath 2012; 20: 83-88.

41 Jacques apple-green birefringence JLaryngOtol 2013; 127: 426-428.

42 Jameson green birefringence ACSChemNeurosci 2012; 3: 807-819.

43 Jowkar apple green birefringence DermatTher 2020; 33: e13278.

44 Kagan green birefringence ProgMolBiolTrSci 2012; 107: 295-325.

45 Kagan Jang characteristic green birefringence MolPharm 2012; 9: 708-717.

46 Kaushik apple-green birefringence SpecCareDent 2016; 36: 104-107.

47 Kieninger ANOMALOUS typical apple-green birefringence, anomalous colours Amyloid 2011; 18: 47-52.

48 Kim apple-green birefringence IntJClinExpPath 2014; 7: 1805-1808.

49 Klingstedt green birefringence Chem 2013; 19: 10179-10192.

50 Kruczak green birefringence PneumAlergPol 2013; 81: 537-541.

51 Kumar Hong green birefringence AnalChem 2017; 89: 9322-9329.

52 Kumar Sengupta classical green birefringence MedJHemInfDis 2013; 5: e2013005.

53 Kurita apple-green birefringence BloodPurif 2015; 40: 146-154.

54 Larsen Kossmann green birefringence, apple-green birefringence KidInt 2014; 86: 378-382.

55 Larsen Walker apple green birefringence KidInt 2010; 77: 816-819.

56 Lee distinctive red-green birefringence ACSChemNeurosci 2019; 10: 2647-2657.

57 Leung apple-green birefringence Blood 2012; 120: 3206-3213.

58 Liu Gilbert apple-green birefringence JVetDiagInv 2012; 24: 1184-1188.

59 Liu Zhang green birefringence, apple-green birefringence EyeLond 2014; 28: 26-33.

60 Long typical apple green birefringence; yellowish green birefringence OphthGenet 2012; 33: 28-33.

61 LoRicco green birefringence JBiolChem 2016; 291: 14045-14055.

62 Luigetti characteristic green birefringence BrainSci 2020; 10: 26.

63 Malyszko MISQUOTE typical apple green birefringence AdvMedSci 2017; 62: 31-38. [Bennhold H. Eine spezifische Amyloidfärbung mit Kongorot. Munch Med Woch 1922; 69: 1537-1538.]

64 Manaa WRONG red-green dichroism SaudJOphth 2017; 31: 180-182.

65 Manoli apple green birefringence MolGenetMetab 2013; 108: 249-254.

66 Mao apple green birefringence Nanosc 2011; 3: 1592-1599.

67 Martini classical apple-green birefringence BMCCardDis 2020; 20: 466.

68 McCarthy green birefringence AmJObsGyn 2016; 215: 464.

69 Mehortra green birefringence JClinDiagRes 2017; 11: WC01-WC05.

70 Meneses typical apple-green birefringence RheumClin 2015; 11: 242-243.

71 Menter MISQUOTE typical apple green birefringence Pathobiol 2017; 84: 49-55. [Divry P, Florkin M. Sur les propriétés optiques de l’amyloïde. C R Sèances Soc Biol 1927; 97: 1808-1810.]

72 Mittal typical red-green birefringence Pathol 2013; 45: 424-426.

73 Mold apple green birefringence IntJEnvResPubH 2019; 16: 16.

74 Muchtar apple green birefringence; vivid green birefringence ActaHaem 2016; 135: 172-190.

75 Nabuurs characteristic green birefringence JAlzDis 2013; 34: 1037-1049.

76 Nakagun green birefringence VetPath 2020; 57: 437-444.

77 Nasir characteristic apple-green birefringence HemodialInt 2020; 24: E1-E4.

78 Navarro characteristic green birefringence JAlzDis 2013; 35: 589-597.

79 Orrego green birefringence, characteristic apple-green birefringence AaceClinCaseRep 2019; 5: e326-e329.

80 Oshima green birefringence JNeurolNeurosPsych 2014; 85: 740-746.

81 Patil apple-green birefringence IranJPath 2016; 11: 448-451.

82 Picken the findings should be described in detail in order to avoid a statement that is not fully correct CurrOpNephHyp 2021; 31: 31.

83 Pihlamaa typical red to green birefringence Amyloid 2016; 23: 225-233.

84 Pinarbasi green SaudJKidDisTrans 2019; 30: 1450-1456.

85 Rajamohamedsait apple-green birefringence MethMolBiol 2012; 849: 411-424.

86 Real de Asua WRONG metachromatic birefringence, classic apple-green birefringence, green–yellow, blue–yellow, and red–green are equally characteristic ClinEpid 2014; 6: 369-377.

87 Rezk WRONG and MISQUOTE pathognomonic apple-green dichroism; pathognomonic green birefringence, pathognomonic apple green birefringence JPathClinRes 2019; e2013005. [Puchtler H, Sweat F, Levine M. On the binding of Congo red by amyloid. J Histochem Cytochem 1962; 10: 355-364.]

88 Russell WRONG red–green dichroism JFishDis 2015; 38: 561-565.

89 Rysalva apple-green birefringence NDT 2019; 34: 1460-1466.

90 Sacsaquispe characteristic apple-green birefringence MedOralPatol 2011; 16: e149-e152.

91 Saha apple-green to yellow-orange birefringence AmJKidDis 2013; 62: 834-838.

92 Saha Theis apple-green birefringence NDT 2011; 26: 2407-2412.

93 Sari Aslani characteristic apple-green birefringence Cureus 2020; 12: e7606.

94 Sarkar MISQUOTE green birefringence MetabBrainDis 2020; 35: 1371-1383. [Divry P. Etude histochimique des plaques seniles. J Belge Neurol Psychiatr 1927; 27: 643-657.]

95 Sedighi red to apple green birefringence AvicJPhytomed 2019; 9: 362-373.

96 Sethi Dasari characteristic apple green-orange birefringence KidIntRep 2018; 3: 1193-1201.

97 Sethi Rajkumar ANOMALOUS apple green birefringence or other anomalous colors JASN 2018; 29: 1810-1823.

98 Sethi Theis apple green-orange birefringence JNeph 2018; 31: 343-350.

99 Sethi Vrana apple-green birefringence KidInt 2012; 82: 226-234.

100 Shanmugam green birefringence BiophysRev 2019; 11: 287-302.

101 Sharma apple-green birefringence CurrOpCardiol 2013; 28: 242-248.

102 Sjolander MISQUOTE and ANOMALOUS anomalous green birefringence Amyloid 2016; 23: 98-108. [Puchtler H, Sweat F, Levine M. On the binding of Congo red by amyloid. J Histochem Cytochem 1962; 10: 355-364. Howie AJ, Brewer DB. Optical properties of amyloid stained by Congo red: history and mechanisms. Micron 2009; 40: 285-301.]

103 Soliman apple-green birefringence RenFail 2019; 41: 850-854.

104 Stefansson MISQUOTE apple-green birefringence Biotech 2012; 52: 2012/04/01. [Howie AJ, Brewer DB. Optical properties of amyloid stained by Congo red: History and mechanisms. Micron 2009; 40: 285-301.]

105 Sueyoshi green birefringence; apple green birefringence HumPath 2011; 42: 1259-1264.

106 Syed pathognomonic apple-green birefringence; characteristic green birefringence EurJGastHepat 2016; 28: 1109-1121.

107 Takanashi characteristic apple-green birefringence Amyloid 2013; 20: 151-155.

108 Tang green birefringence JAgrFoodChem 2010; 58: 11058-11066.

109 Tomova apple-green birefringence Cureus 2020; 12: e8777.

110 Trikha apple green birefringence Cornea 2011; 30: 716-717.

111 von Mikecz green birefringence Nucleus 2014; 5: 311-317.

112 Wang Li apple-green birefringence BMCGastro 2013; 13: 71.

113 Wang Huang characteristic yellow-green birefringence JBiomolStrDyn 2012; 30: 160-169.

114 Winter MISQUOTE characteristic green birefringence Proteom 2017; 17: 2017/10/11. [Puchtler H, Sweat F, Levine M. On the binding of Congo red by amyloid. J Histochem Cytochem 1962; 10: 355-364. Howie AJ, Brewer DB. Optical properties of amyloid stained by Congo red: history and mechanisms. Micron 2009; 40: 285-301. Howie AJ, Brewer DB, Howell D, Jones AP. Physical basis of colors seen in Congo red-stained amyloid in polarized light. Lab Invest 2008; 88: 232-242.]

115 Wu MISQUOTE characteristic apple-green birefringence BiophysJ 2012; 103: 550-557. [Howie AJ, Brewer DB, Howell D, Jones AP. Physical basis of colors seen in Congo red-stained amyloid in polarized light. Lab Invest 2008; 88: 232-242.]

116 Yepez apple green birefringence CaseRepOphth 2020; 11: 287-292.

117 Zhao apple-green birefringence JAlzDis 2015; 5: 177.
