# Supplementary material for: Systematic review of accuracy of reporting of Congo red-stained amyloid in 2010–2020 compared with earlier
Source: Ann Med. 2022 Sep 18;54(1):2511–6. doi: 10.1080/07853890.2022.2123558 (PMC9518257; doi:10.1080/07853890.2022.2123558)
Supplement: Supplemental Material [file IANN_A_2123558_SM2105.docx]

**Systematic review of accuracy of reporting of Congo red-stained amyloid in 2010-2020 compared with earlier**

**Supplement 1: papers with descriptions and figures**

Alexander J Howie and Mared P Owen-Casey

Each entry is in this order: Number in this Supplement; name of first author; if relevant, WRONG EXPRESSION (dichroism or metachromasia or fluorescence), or MISQUOTE of a reference, or ANOMALOUS if mentioned; description of colour(s) in text; reference; [if relevant, misquoted reference]; COLOUR(S) IN IMAGE(S), with **GREEN** alone, if seen; DISCREPANCY or AGREE; number of images with **NO GREEN**

**n = 257**

1 Abdallah WRONG EXPRESSION characteristic apple-green dichroism AmJCaseRep 2012; 13: 102-105. GREEN, YELLOW; DISCREPANCY X1

2 Accardi characteristic green birefringence, apple green birefringence CaseRepHematol 2018; 9840405. GREEN, WHITE, YELLOW, ORANGE, 3b BLUE, YELLOW; DISCREPANCY X2 [**1 NO GREEN**]

3 Adachi MISQUOTE blue-green birefringence BMCNephrol 2020; 21: 195. [Sipe JD, Benson MD, Buxbaum JN, et al. Amyloid fibril protein nomenclature: 2012 recommendations from the nomenclature Committee of the International Society of amyloidosis. Amyloid 2012; 19: 167-170.] GREEN, RED; DISCREPANCY X1

4 Adamo typical apple-green birefringence JOralMaxSurg 2020; 78: 1572-1582. **GREEN**; AGREE X1

5 Ahn apple-green birefringence ClinEndosc 2018; 51: 285-288. GREEN, YELLOW; DISCREPANCY X1

6 Akasaki green birefringence; apple green birefringence ArthRheum 2015; 67: 2097-2107. 2 **GREEN**; AGREE X2

7 Alexander MISQUOTE green, yellow, and red/ orange birefringence AmJKidDis 2018; 72: 325-336. [Howie AJ, Brewer DB. Optical properties of amyloid stained by Congo red: history and mechanisms. Micron 2009; 40: 285-301. Howie AJ. "Green (or apple-green) birefringence" of Congo red-stained amyloid. Amyloid 2015; 22: 205-206.] GREEN, RED; TOO DARK; ORANGE, RED; DISCREPANCY X3 [**2 NO GREEN**]

8 Altwairgi classical / characteristic apple-green birefringence IntJHealthSci 2011; 5: 181-185. **GREEN**; AGREE X1

9 Andeen apple-green birefringence CJASN 2019; 14: 1741-1750. GREEN, ORANGE; DISCREPANCY X1

10 Angiero apple-green birefringence; apple-green and red birefringence AnticancRes 2010; 30: 3009-3014. **GREEN**; DISCREPANCY X1

11 Araki-Sasaki apple-green birefringence ClinOphth 2014; 8: 2115-2119. YELLOW, ORANGE, WHITE; DISCREPANCY X1 [**1 NO GREEN]**

12 Aranda-Souza apple-green birefringence; green birefringence Toxicon 2019; 157: 93-100. **GREEN**; AGREE X1

13 Audet characteristic yellow–green birefringence Histopath 2012; 61: 610-619. GREEN, YELLOW; YELLOW; YELLOW; AGREE X1, DISCREPANCY X2 [**2 NO GREEN**]

14 Babburi apple green birefringence JClinDiagRes 2013; 7: 3094-3095. GREEN, YELLOW; DISCREPANCY x1

15 Baresic green birefringence, characteristic apple-green birefringence Lupus 2015; 24: 1546-1551. **GREEN**; AGREE X1

16 Barran-Berdon MISQUOTE and ANOMALOUS anomalous yellow-green or orange colors SciRep 2020; 10: 5138. [Howie AJ, Brewer DB. Optical properties of amyloid stained by Congo red: History and mechanisms. Micron 2009; 40: 285-301. Howie AJ, Brewer DB, Howell D, Jones AP. Physical basis of colors seen in Congo red-stained amyloid in polarized light. Lab Invest 2008; 88: 232-242.] YELLOW, ORANGE X7, ORANGE X5; DISCREPANCY X12 [**12 NO GREEN**]

17 Basu yellow and green birefringence HeadNeckPathol 2020; 14: 808-816. GREEN, YELLOW; AGREE X1

18 Bell apple-green birefringence CaseRepPathol 2020; 8830763. YELLOW, BLUE; DISCREPANCY X1 [**1 NO GREEN**]

19 Bhavsar apple-green birefringence ActaCytol 2011; 55: 296-301. YELLOW; DISCREPANCY X1 [**1 NO GREEN**]

20 Bock green birefringence VetPathol 2011; 48: 906-910. GREEN, YELLOW; DISCREPANCY X1

21 Bowen characteristic apple-green birefringence CaseRepNephrol 2012; 593460. ORANGE, WHITE; DISCREPANCY X1 [**1 NO GREEN**]

22 Bucci characteristic apple-green birefringence JMedCaseRep 2014; 8: 198. GREEN, RED; DISCREPANCY X1

23 Buhimschi green birefringence SciTranslMed 2014; 6: 245ra92. GREEN, YELLOW; DISCREPANCY X1

24 Burns green birefringence, apple-green birefringence JCompPathol 2017; 157: 136-140. GREEN, RED; DISCREPANCY X1

25 Burra characteristic apple-green birefringence JMolecBiol 2021; 433: 166732. 2 **GREEN**, 1 GREEN, YELLOW; AGREE X2, DISCREPANCY X1

26 Burrough apple-green birefringence VetPathol 2012; 49: 616-620. RED, BLUE, YELLOW; DISCREPANCY X1 [**1 NO GREEN**]

27 Buxbaum MISQUOTE green birefringence JMolBiol 2012; 421: 142-159. [Ladewig P. Double-refringence of the amyloid-Congo-red-complex. Nature 1945; 156: 81-82.] **GREEN**; AGREE X1

28 Byers apple-green birefringence ExpMolPathol 2018; 104: 151-154. GREEN, YELLOW; DISCREPANCY X1

29 Carnaccini apple-green birefringence AvianDis 2016; 60: 33-42. GREEN, RED; BLUE, YELLOW; DISCREPANCY X2 [**1 NO GREEN**]

30 Castrodad-Rodriguez apple green birefringence ModPathol 2020; 33: 1581-1588. RED, WHITE; DISCREPANCY X1 [**1 NO GREEN**]

31 Chabert typical red-green birefringence KidneyInt 2019; 96: 628-641. 2 RED, GREEN; 2 RED, GREEN, YELLOW; AGREE X2, DISCREPANCY X2

32 Chatterjee typical apple green birefringence DentResJ 2017; 14: 293-296. GREEN, YELLOW; DISCREPANCY X1

33 Chen Sun apple-green birefringence Med 2019; 98: e16830. GREEN, YELLOW, WHITE; DISCREPANCY X1

34 Chen Wang pathognomonic / typical apple-green birefringence Oncotarg 2018; 9: 24283-24290 GREEN, RED, WHITE; DISCREPANCY X1

35 Chen Hua apple-green IntJNanomed 2018; 13: 2477-2489. GREEN, YELLOW, RED; GREEN, YELLOW; DISCREPANCY X2

36 Chen Peng apple-green birefringence ACSOmega 2020; 5: 11677-11686. 6 **GREEN**; 2 GREEN, YELLOW; 3 GREEN, YELLOW, RED; AGREE X6, DISCREPANCY X5

37 Chen Tang typical ‘apple-green’ birefringence*, green birefringence JPeptSci 2013; 19: 708-716. 3 RED, BLUE; 1 YELLOW, BLUE; DISCREPANCY X4 [**4 NO GREEN**]

38 Chhoda apple-green birefringence ACGCaseRepJ 2018; 5: e24. GREEN, YELLOW; DISCREPANCY X1

39 Cirulis apple-green birefringence PulmCirc 2016; 6: 244-248. RED, YELLOW; DISCREPANCY X1 [**1 NO GREEN**]

40 Clement apple-green birefringence HumanPathol 2014; 45: 1766-1772. 2 WHITE; DISCREPANCY X2 [**2 NO GREEN**]

41 Clos green birefringence, apple-green birefringence JAmAcadDerm 2011; 65: 1023-1031. 2 **GREEN**; AGREE X2

42 Coppock green birefringence, characteristic orange coloration with green birefringence JASC 2020; 9: 173-176. GREEN, YELLOW, ORANGE; DISCREPANCY X2

43 Correa WRONG EXPRESSION characteristic red-green birefringence and dichroism BMCOphthal 2015; 15: 77. RED, YELLOW; DISCREPANCY X1 [**1 NO GREEN**

44 Costache characteristic dichroism and apple-green birefringence RomJMorphEmbryol 2017; 58: 201-206. WHITE; DISCREPANCY X1 [**1 NO GREEN**]

45 Dasari green-gold birefringence Chembiochem 2011; 12: 407-423. 1 GREEN, YELLOW, ORANGE; 1 **GREEN**; DISCREPANCY X2

46 Daude gold/green structures JNeurochem 2010; 113: 92-104. 3 ORANGE, YELLOW; 2 YELLOW; 1 ORANGE; DISCREPANCY X6 [**6 NO GREEN**]

47 den Braber-Ymker apple green NeurogastMotil 2018; 30: e13469. **GREEN**; AGREE X1

48 Desport typical / characteristic apple-green birefringence OrphJRareDis 2012; 7: 54. YELLOW, BLUE; DISCREPANCY X1 [**1 NO GREEN**]

49 Dezfulian green birefringence IntImmunopharm 2018; 59: 106-112. POOR IMAGES; DISCREPANCY x3 [**3 NO GREEN**]

50 Di Crescenzo WRONG EXPRESSION metachromatic green, apple-green birefringence, typical green birefringence, red with apple-green birefringence BMCSurg 2013; 13: S43. POOR IMAGE; DISCREPANCY x1 [**1 NO GREEN**]

51 Diaz-Flores yellow-green birefringence DiagPathol 2017; 12: 58. 1 BLUE, YELLOW; 1 GREEN, YELLOW; AGREE X1, DISCREPANCY X1 [**1 NO GREEN**]

52 Doe-Williams green birefringence, characteristic apple-green birefringence CaseRepGastr 2020; 14: 271-278. YELLOW; DISCREPANCY X1 [**1 NO GREEN**]

53 Dogan MISQUOTE and ANOMALOUS characteristic anomalous color spectrum of orange and green AnnRevPath 2017; 12: 277-304. [Howie AJ. Diagnosis of amyloid using Congo red. In Picken MM, Herrera GA, Dogan A. Amyloid and Related Disorders. Surgical Pathology and Clinical Correlations. New York: Humana. 2nd ed. 2015: 197-212. Howie AJ, Brewer DB. Optical properties of amyloid stained by Congo red: history and mechanisms. Micron 2009; 40: 285-301.] GREEN, ORANGE, RED; GREEN, YELLOW, WHITE; GREEN, ORANGE, YELLOW, WHITE; DISCREPANCY X3

54 Doganavsargil MISQUOTE apple-green birefringence, the gold standard for definitive diagnosis since it was first described by Kyle in 1927. WorldJGast 2015; 21: 1827-1837. [Kyle RA. Amyloidosis: a convoluted story. Br J Haematol 2001; 114: 529-538.] 4 **GREEN**; AGREE X4

55 Du green birefringence; apple green PLOSone 2015; 10: e0135499. ORANGE; DISCREPANCY X1 [**1 NO GREEN**]

56 Ebenezer MISQUOTE apple-green birefringence; yellow–green birefringence AnnNeurol 2017: 82: 44-56. [Howie AJ, Brewer DB, Howell D, Jones AP. Physical basis of colors seen in Congo red-stained amyloid in polarized light. Lab Invest 2008; 88: 232-242.] 1 RED, YELLOW; 3 ORANGE, YELLOW; 1 GREEN, YELLOW, WHITE; 1 ORANGE, YELLOW, WHITE; 1 GREEN, YELLOW, ORANGE, WHITE; DISCREPANCY X7 [**5 NO GREEN**]

57 El-Meanawy MISQUOTE apple-green birefringence, green birefringence DiagPathol 2019; 14: 57. [Howie AJ. Apple-green birefringence? The Bulletin of the Royal College of Pathologists 2008; 144:263-266.] 1 **GREEN**; 3 GREEN, YELLOW; 1 GREEN, YELLOW, ORANGE; 1 RED, YELLOW; 2 GREEN, YELLOW, RED; AGREE X1**,** DISCREPANCY X 7 [**1 NO GREEN**]

58 Elkins yellow-green birefringence DiagCytopath 2012; 40: E126-130. GREEN, YELLOW, RED; DISCREPANCY X1

59 Esmat apple-green birefringence LaserSurgMed 2015; 47: 388-395. ORANGE, RED; DISCREPANCY X1 [**1 NO GREEN**]

60 Exley apple green birefringence Heliyon 2020; 6: e03839. 5 **GREEN**; AGREE X5

61 Factor characteristic apple-green birefringence DiagCytopath 2012; 40: E114-117. GREEN, RED; DISCREPANCY X1

62 Farci apple green birefringence CardiovPath 2019; 41: 21-23. GREEN, RED; DISCREPANCY X1

63 Fernandes apple green birefringence JCutPath 2011; 38: 827-831. GREEN, YELLOW, RED; DISCREPANCY X1

64 Fortin characteristic apple-green birefringence VetMedSci 2018; 4: 63-70. RED, YELLOW; DISCREPANCY X1 [**1 NO GREEN**]

65 Gambella typical / characteristic green birefringence EndocPath 2020; 12: 12. 1 GREEN, YELLOW, WHITE; 1 GREEN, YELLOW, WHITE; DISCREPANCY X2

66 Garcia apple green birefringence ArchSocEspOftal 2020; 95: 244-247. GREEN, BLUE, YELLOW, RED; DISCREPANCY X1

67 Gerasimova yellow to apple-green birefringence IntJMolSci 2019; 20: 7. GREEN, YELLOW, ORANGE; DISCREPANCY X1

68 Gillmore pathognomonic green birefringence NatRevNeph 2013; 9: 574-586. 1 **GREEN**; 1 YELLOW, BLUE; AGREE X1, DISCREPANCY X1 [1 **NO GREEN**]

69 Ginat typical apple-green birefringence HeadNeckPath 2018; 12: 127-130. YELLOW, RED; DISCREPANCY X1 [1 **NO GREEN**]

70 Gioeva green birefringence; typical apple-green birefringence Amyloid 2013; 20: 1-6. YELLOW; DISCREPANCY X1 [1 **NO GREEN**]

71 Gjeorgjievski classic apple-green birefringence CaseRepGastMed 2015; 320120. 1 GREEN, RED; 1 GREEN, YELLOW, RED; DISCREPANCY X2

72 Gouvea apple-green color JOralPathMed 2012; 41: 178-185. 1 GREEN, YELLOW; 1 BLUE, YELLOW; DISCREPANCY X2 [1 **NO GREEN**]

73 Greunz apple green birefringence JZooWildMed 2020; 51: 202-209. GREEN, YELLOW, RED; DISCREPANCY X1

74 Gu typical apple-green birefringence JZhieUnivSci 2014; 15: 92-99. YELLOW, RED; DISCREPANCY X1 [**1 NO GREEN]**

75 Gupta characteristic apple-green birefringence Protoplasm 2020; 257: 1259-1276. ORANGE, YELLOW; DISCREPANCY X1 [1 **NO GREEN**]

76 Gusel'nikova WRONG EXPRESSION reddish and apple-green fluorescence was observed by the polarized light analysis; apple-green birefringence EurJHistochem 2018; 62: 2870. GREEN, RED; AGREE X2

77 Habiba apple green birefringence JAlzDis 2020; 76: 1135-1150. 1 RED, WHITE; 1 WHITE; DISCREPANCY X2 [2 **NO GREEN**]

78 Habibi apple-green birefringence AvianPath 2017; 46: 497-505. YELLOW; DISCREPANCY X1 [**1 NO GREEN**]

79 Hahn typical green-yellow-orange birefringence Amyloid 2017; 24: 78-86. YELLOW; DISCREPANCY X1 [**1 NO GREEN**]

80 Hammami yellow-green double refraction EurAnnOtorhin 2010; 127: 83-85. GREEN, YELLOW, RED, WHITE; DISCREPANCY X1

81 Heys characteristic apple green birefringence EnvSciTech 2017; 51: 8672-8681. 4 **GREEN**; AGREE X4

82 Hiew characteristic apple green birefringence ActaBiomat 2016; 46: 41-54. 6 GREEN, RED; DISCREPANCY X6

83 Hirayama apple green birefringence VetPathol 2017; 54: 218-221. **GREEN**; AGREE X1

84 Ho apple green birefringence BrainPath 2014; 24: 25-32. GREEN, RED; DISCREPANCY X1

85 Hoane green birefringence ToxPath 2016; 44: 687-704. GREEN, RED; DISCREPANCY X1

86 Hokama unique apple-green birefringence WorldJGastEndosc 2011; 3: 157-161. **GREEN**; AGREE X1

87 Hoscheit apple-green birefringence CaseRepGast 2018; 12: 317-321. YELLOW; DISCREPANCY X1 [1 **NO GREEN**]

88 Hsiao WRONG EXPRESSION characteristic red-green birefringence and dichroism, apple-green birefringence ClinChimAct 2019; 494: 112-115. GREEN, YELLOW; DISCREPANCY X1

89 Hung typical apple-green birefringence JChinMedAss 2010; 73: 161-165. YELLOW, WHITE; DISCREPANCY X1 [**1 NO GREEN**]

90 Ichimata apple-green birefringence PathInternat 2019; 69: 235-240. 3 GREEN, YELLOW; DISCREPANCY X3

91 Ida apple-green birefringence EndocPath 2013; 24: 149-155. YELLOW, RED; DISCREPANCY X1 [**1 NO GREEN**]

92 Inayat characteristic apple-green birefringence BMJCaseRep 2019; 12: 1. GREEN, YELLOW; DISCREPANCY X1

93 Ishii apple-green birefringence AnnNucMed 2012; 26: 522-526. GREEN, YELLOW; DISCREPANCY X1

94 Itagaki green birefringence CardiovPath 2020; 46: 107191. **GREEN**; AGREE X1

95 Itakura apple-green birefringence JBiolChem 2015; 290: 26072-26087. GREEN, RED; DISCREPANCY X1

96 Jacob green gold birefringence, greenish-yellow birefringence SciRep 2016; 6: 23370. GREEN, YELLOW, ORANGE; DISCREPANCY X1

97 Jagusiak MISQUOTE and ANOMALOUS apple-green effect; presented effects are up to now explained on the physical grounds, in particular by anomalous dispersion of the refractive index ActaBiochPol 2019; 66: 39-46. [Howie AJ, Brewer DB, Howell D, Jones AP Physical basis of colors seen in Congo red-stained amyloid in polarized light. Lab Invest 2008; 88: 232-242. Howie AJ, Brewer DB Optical properties of amyloid stained by Congo red: history and mechanisms. Micron 2009; 40: 285-301. Benditt EP, Eriksen N, Berglund C. Congo red dichroism with dispersed amyloid fibrils, an extrinsic cotton effect. Proc Natl Acad Sci USA 1970; 66: 1044-1051.] GREEN, YELLOW, RED; DISCREPANCY X1

98 Jamet typical apple-green birefringence AmJSurgPath 2015; 39: 1035-1044. BLUE, YELLOW; DISCREPANCY X1 [1 **NO GREEN**]

99 Jana typical green-yellow birefringence AngewChemInt 2016; 55: 15287-15291. YELLOW; DISCREPANCY X1 [1 **NO GREEN**]

100 Javidiparsijani characteristic apple green birefringence ArchPathLabMed 2020; 16: 16. 3 GREEN, YELLOW, RED; 1 GREEN, YELLOW, RED, WHITE; 1 **GREEN**; DISCREPANCY X4, AGREE X1

101 Jung green birefringence, red-apple green birefringence Cornea 2014; 33: 1324-1331. GREEN, YELLOW; DISCREPANCY X1

102 Kadota green polarization VetPath 2020; 57: 658-665. **GREEN**, 1 GREEN, YELLOW, RED; DISCREPANCY X1, AGREE X1

103 Kalhor green-blue light Langmuir 2011; 27: 10776-10784. 2 BLUE, YELLOW, WHITE; 2 BLUE, WHITE; DISCREPANCY X4 [**4 NO GREEN**]

104 Kaliyamurthi green birefringence, characteristic apple green birefringence JBioSci 2012; 37: 1017-1027. 2 YELLOW, 2 WHITE; DISCREPANCY X4 [4 **NO GREEN**]

105 Kalle apple-green birefringence JLabPhys 2018; 10: 226-231. YELLOW; DISCREPANCY X1 [1 **NO GREEN**]

106 Kamata green birefringence JMedCaseRep 2012; 6: 231. 2 **GREEN**; AGREE X2

107 Kamiie characteristic green birefringence VetPath 2017; 54: 111-118. 3 GREEN, RED; DISCREPANCY X3

108 Kanno apple-green birefringence RespCaseRep 2018; 6: e00305. **GREEN**; AGREE X1

109 Katagiri red to green birefringence Biochem 2010; 49: 5909-5918. 1 RED; 1 GREEN, YELLOW; 1 **GREEN**; 1 TOO DARK; 1 YELLOW; DISCREPANCY X5 [**3 NO GREEN**]

110 Kato apple-green birefringence JMedCaseRep 2017; 11: 216. GREEN, YELLOW, ORANGE; DISCREPANCY X1

111 Katzman apple green birefringence AmJDermpath 2018; 40: 527-530. YELLOW, GREEN, BLUE; DISCREPANCY X1

112 Kaur yellow-green birefringence FrontMicrobiol 2018; 9: 1934. GREEN, YELLOW; AGREE X1

113 Kawasaki Tsukiyama yellow-green birefringence SciRep 2019; 9: 10636. 4 ORANGE; DISCREPANCY X4 [**4 NO GREEN**]

114 Kawasaki Yaji green-yellow birefringence CellMolNeurobiol 2018; 38: 1039-1049. 3 ORANGE; DISCREPANCY X3 [**3 NO GREEN**]

115 Kelsey apple-green birefringence IntJWomDerm 2016; 2: 18-22. BLUE, YELLOW, GREEN, ORANGE; DISCREPANCY X1

116 Kershaw green birefringence, typical apple-green birefringence VetOphth 2011; 14: 88-92. GREEN, YELLOW; DISCREPANCY X1

117 Khan apple green birefringence AnnDiagPath 2019; 40: 1-6. 3 GREEN, YELLOW; DISCREPANCY X3

118 Kimmich MISQUOTE typical apple-green birefringence Amyloid 2017; 24: 52-59. [Sipe JD, Benson MD, Buxbaum JN, et al. Amyloid fibril proteins and amyloidosis: chemical identification and clinical classification International Society of Amyloidosis 2016 Nomenclature Guidelines. Amyloid 2016; 23: 209-213.] 2 GREEN, ORANGE; 1 GREEN, YELLOW; DISCREPANCY X3

119 Klaassen green CardiovPath 2017; 29: 19-22. **GREEN**; AGREE X1

120 Kluve-Beckerman green or red Amyloid 2011; 18: 136-146. 1 BLUE, RED; 2 GREEN, RED; 1 **GREEN**; DISCREPANCY X1, AGREE X3 [**1 NO GREEN**]

121 Kogler green birefringence, apple green birefringence BMJCaseRep 2020; 13: 8. RED, YELLOW, ORANGE; DISCREPANCY X1 [1 **NO GREEN**]

122 Kosolapova MISQUOTE green birefringence, green or yellow birefringence Biomol 2019; 9: 4. [Howie AJ, Brewer DB. Optical properties of amyloid stained by Congo red: History and mechanisms. Micron 2009; 40: 285-301.] 5 GREEN, YELLOW; DISCREPANCY X5

123 Krishna apple-green birefringence JOP 2013; 14: 283-285. GREEN, YELLOW, RED; DISCREPANCY X1

124 Kumar characteristic apple green birefringence HeadNeckPath 2016; 10: 379-383. GREEN, YELLOW, RED; DISCREPANCY X1

125 Kumari apple yellow-green birefringence JBiolChem 2020; 295: 3466-3484. GREEN, YELLOW; AGREE X1

126 Labour characteristic green birefringence ActaBiomat 2016; 37: 38-49. 2 GREEN, RED; DISCREPANCY X2

127 Lee apple green birefringence JMedCaseRep 2016; 10: 68. GREEN, RED; DISCREPANCY X1

128 Levine apple-green birefringence OphthPlastReconSurg 2017; 33: e86-e88. GREEN, YELLOW, RED; DISCREPANCY X1

129 Li Li apple-green birefringence, characteristic yellow-green birefringence FrontNeur 2017; 8: 368. 1 GREEN, RED; 1 GREEN, YELLOW, RED; DISCREPANCY X2

130 Li Huang apple-green birefringence PLOSone 2017; 12: e0185078. 1 BLUE, YELLOW; 2 RED, GREEN; 1 GREEN, RED, YELLOW; DISCREPANCY X4 [1 **NO GREEN**]

131 Lin Mao characteristic apple-green birefringence DiagPath 2015; 10: 174. RED, GREEN; DISCREPANCY X1

132 Liu Zhao apple-green birefringence PNASUSA 2012; 109: 20913-20918. 2 GREEN, RED; 1 GREEN, YELLOW; DISCREPANCY X3

133 Liu Lan characteristic green birefringence IntJClinExpPath 2014; 7: 7795-7800. 1 **GREEN**, 2 GREEN, RED, 1 GREEN, YELLOW, RED; DISCREPANCY X3; AGREE X1

134 Louros apple-green birefringence PLOSone 2013; 8: e73258. 3 GREEN, YELLOW, ORANGE; DISCREPANCY X3

135 Louros apple-green birefringence FEBSLett 2014; 588: 52-57. GREEN, YELLOW, RED; DISCREPANCY X1

136 Lytle characteristic apple-green birefringence PathResPract 2019; 215: 152699. BLUE, GREEN, YELLOW; DISCREPANCY X1

137 M Colombat green birefringence, apple-green birefringence KidneyInt 2020; 98: 195-208. 1 **GREEN**; 3 RED, YELLOW, WHITE; 1 GREEN, RED; 1 GREEN, YELLOW; DISCREPANCY X 5, AGREE X1 [3 **NO GREEN**]

138 Machado apple-green birefringence Amyloid 2013; 20: 52-55. GREEN, YELLOW; DISCREPANCY X1

139 Maeda emerald-green birefringence, green birefringence ExpAnim 2016; 65: 427-436. 6 TOO FAINT; DISCREPANCY X6 [**6 NO GREEN**]

140 Maiti apple-green birefringence HistoCellBiol 2016; 146: 609-625. 4 **GREEN**; AGREE X4

141 Maity typical green-gold birefringence OrgBiomolChem 2011; 9: 3787-3791. GREEN, YELLOW, RED; DISCREPANCY X1

142 Manur apple-green birefringence DiagCytopath 2018; 46: 522-524. WHITE, RED; DISCREPANCY X1 [**1 NO GREEN**]

143 Marcus green birefringence, apple green birefringence AmJClinPath 2012; 138: 590-593. WHITE, RED; DISCREPANCY X1 [**1 NO GREEN]**

144 Markande apple green birefringence Biofoul 2016; 32: 1153-1162. **GREEN**; AGREE X1

145 Martinez apple-green birefringence JZooWildMed 2019; 50: 147-158. GREEN, RED; DISCREPANCY X1

146 Matsuo apple-green birefringence ClinOralInvest 2016; 20: 1913-1920. GREEN, YELLOW, RED, WHITE; DISCREPANCY X1

147 Mawby green birefringence, apple green birefringence JFMSOpenRep 2018; 4: 2055116918817993. 1 WHITE; 1 GREEN, YELLOW, RED; DISCREPANCY X2 [1 **NO GREEN**]

148 McFarlane apple green birefringence BMJCaseRep 2018; 2018/07/12. BLUE, YELLOW, RED; DISCREPANCY X1 [1 **NO GREEN**]

149 Mei typical apple-green birefringence BMCNephrol 2020; 21: 140. WHITE; DISCREPANCY X1 [1 **NO GREEN**]

150 Mendoza apple-green birefringence ProcBaylUnivMedCent 2010; 23: 139-141. YELLOW; DISCREPANCY X1 [1 **NO GREEN**]

151 Metkar apple red/green birefringence, apple-green birefringence IntJBiolMacro 2020; 163: 128-134. GREEN, RED; AGREE X1

152 Michael Otto red-to-apple green shift (birefringence), apple-green birefringence ExpEyeRes 2014; 119: 44-53. 1 **GREEN,** 1 WHITE; DISCREPANCY X1, AGREE X1 [**1 NO GREEN**]

153 Michael Rosandic apple-green birefringence ExpEyeRes 2013; 106: 5-13. 2 **GREEN**, 1 WHITE, 1 YELLOW, 1 GREEN, YELLOW, 1 GREEN, ORANGE; DISCREPANCY X4, AGREE X2 [2 **NO GREEN**]

154 Millucci Ghezzi characteristic green birefringence SciWorldJ 2012; 293863. 2 YELLOW; DISCREPANCY X2 [2 **NO GREEN**]

155 Millucci Spreafico green birefringence BiochimBiophysActa 2012; 1822: 1682-1691. 4 WHITE; 3 RED, YELLOW; 1 GREEN, WHITE, YELLOW, ORANGE; 1 GREEN, YELLOW, RED; 4 RED; 1 GREEN, RED; DISCREPANCY X14 [11 **NO GREEN**]

156 Minamimoto red–green birefringence AmJNucMedMolIm 2013; 3: 261-271. YELLOW, WHITE; DISCREPANCY X1 [**1 NO GREEN**]

157 Miura apple green birefringence GerGerInt 2019; 19: 1054-1062. **GREEN**; AGREE X1

158 Mold classical apple-green birefringence IntJEnvResPubH 2019; 16: 24. 4 **GREEN**, GREEN + YELLOW 1, GREEN + RED 2; DISCREPANCY X3, AGREE X4

159 Monge-Morera typical / characteristic green birefringence Biomacromol 2020; 21: 2218-2228. 2 GREEN, YELLOW, ORANGE; DISCREPANCY X2

160 Mori green birefringence SurgCaseRep 2019; 5: 31. YELLOW, RED; DISCREPANCY X1 [**1 NO GREEN**]

161 Muller apple-green birefringence CardiovPath 2013; 22: 195-202. 1 GREEN, RED; 1 YELLOW, RED; DISCREPANCY X2 [1 **NO GREEN**]

162 Musat apple-green birefringence MolClinOnc 2020; 12: 258-262. GREEN, BLUE, YELLOW; DISCREPANCY X1

163 Nalcacioglu apple-green birefringence IntJRheumDis 2018; 21: 552-559. YELLOW, RED; DISCREPANCY X1 [**1 NO GREEN**]

164 Nam apple green birefringence AnnDerm 2017; 29: 79-82. TOO PALE; DISCREPANCY X1 **[1 NO GREEN]**

165 Nasr green birefringence, apple-green birefringence JASN 2017; 28: 439-445. GREEN, YELLOW; DISCREPANCY X1

166 Nasr apple-green birefringence JASN 2017; 28: 431-438. BLUE, YELLOW, ORANGE; DISCREPANCY X1 [1 **NO GREEN**]

167 Nasr Said diagnostic apple green birefringence KidInt 2013; 83: 463-470. GREEN, ORANGE; DISCREPANCY X1

168 Neo-Suzuki green birefringence VetClinPath 2017; 46: 331-336. 1 WHITE, RED; 1 GREEN, YELLOW, ORANGE; DISCREPANCY X2 [1 **NO GREEN**]

169 Nesic WRONG EXPRESSION fluorescent yellow–green under the polarized light VetQ 2017; 37: 1-7. 2 GREEN, ORANGE; DISCREPANCY X2

170 Nevo typical apple-green birefringence Urol 2020; 145: 253-257. 2 GREEN, YELLOW, RED; DISCREPANCY X2

171 Nieva classic apple-green birefringence ChemBiol 2011; 18: 920-927. GREEN, YELLOW, WHITE; DISCREPANCY X1

172 Nobakht typical green birefringence IranBiomedJ 2011; 15: 51-58. GREEN, ORANGE; DISCREPANCY X1

173 Obici green birefringent material KidInt 2016; 90: 479-481. GREEN, YELLOW; DISCREPANCY X1

174 Oldak characteristic apple-green birefringence Cornea 2014; 33: 294-299. GREEN, YELLOW; DISCREPANCY X1

175 Oli MISQUOTE green birefringent, yellowish apple-green, yellowish-green birefringence Microbiol 2012; 158: 2903-2916. [Howie AJ, Brewer DB, Howell D, Jones AP Physical basis of colors seen in Congo red-stained amyloid in polarized light. Lab Invest 2008; 88: 232-242.] 3 GREEN, YELLOW; 4 GREEN, YELLOW, ORANGE; 2 YELLOW; 3 **GREEN**; 3 POOR; DISCREPANCY X12, AGREE X3 [5 **NO GREEN]**

176 Oliveira birefringent green coloration AnBrasDerm 2012; 87: 119-122. **GREEN**; AGREE X1

177 Ostevik characteristic green birefringence, apple green birefringence ActVetScand 2014; 56: 50. YELLOW, ORANGE; DISCREPANCY X1 [1 **NO GREEN**]

178 Ostrow apple-green birefringence MuscNerv 2012; 45: 755-761. 2 YELLOW, RED; 2 WHITE, RED; DISCREPANCY X4 [4 **NO GREEN**]

179 Pacifico a green color ChemResTox 2014; 27: 611-626. 6 **GREEN**; AGREE X6

180 Paikar typical green-gold birefringence OrgBiomolChem 2017; 15: 4218-4225. 2 GREEN, YELLOW, RED; DISCREPANCY X2

181 Papa apple green birefringence RheumDisClinNAm 2018; 44: 585-603. GREEN, YELLOW; DISCREPANCY X1

182 Patel characteristic yellow–green birefringence Cornea 2010; 29: 1215-1222. **GREEN;** DISCREPANCY X1

183 Paul golden-green birefringence ChemAEurJ 2020; 26: 16486-16496. 2 YELLOW, GREEN, RED; DISCREPANCY x2

184 Petruzziello WRONG EXPRESSION a metachromatic green color; apple-green birefringence LeukLymph 2011; 52: 2304-2307. 2 GREEN, RED; 1 GREEN, YELLOW, RED; DISCREPANCY X3

185 Picken apple-green birefringent ArchPathLabMed 2010; 134: 545-551. GREEN, YELLOW; DISCREPANCY X1

186 Picken yellow-green birefringence ActaHem 2020; 143: 322-334. 2 **GREEN**, 1 GREEN, RED; DISCREPANCY X3

187 Pinto apple-green birefringence AdvAnatPath 2013; 20: 61-67. 1 BLUE, GREEN, YELLOW; 1 BLUE; 1 BLUE, YELLOW; 1 GREEN, RED, YELLOW; 1 **GREEN**; DISCREPANCY X4, AGREE X1 [2 **NO GREEN**]

188 Podduturi apple-green birefringence ProcBaylUnivMedC 2013; 26: 387-389. YELLOW, ORANGE; DISCREPANCY X1 [1 **NO GREEN**]

189 Qu apple green birefringence NephCarlt 2010; 15: 102-107. **GREEN**; AGREE X1

190 Raghunathan apple-green birefringence JClinMedRes 2017; 9: 654-658. GREEN, ORANGE; DISCREPANCY X1

191 Rahman typical apple-green birefringence BMJCaseRep 2011; 2011/01/01 **GREEN**; AGREE X1

192 Raivio red-green birefringence Amyloid 2016; 23: 46-50. GREEN, YELLOW, ORANGE; DISCREPANCY X1

193 Rajagopal apple-green birefringence BMJCaseRep 2018; 11: 2018/12/24. YELLOW, BLUE; YELLOW, GREEN; DISCREPANCY X2 [**1 NO GREEN**]

194 Ran apple-green birefringence ExpEyeRes 2018; 169: 13-19. POOR; DISCREPANCY X1 [1 **NO GREEN**]

195 Rancati apple-green birefringence ForensSciInt 2018; 289: 150-153. **GREEN**; AGREE X1

196 Richey green-gold birefringence AmJPath 2019; 2019/02/09. GREEN, YELLOW; AGREE X1

197 Roden apple-green birefringence HumPath 2010; 41: 1040-1045. YELLOW; DISCREPANCY X1 [1 **NO GREEN**]

198 Said diagnostic apple-green birefringence CJASN 2013; 8: 1515-1523. 2 GREEN, YELLOW, ORANGE; 1 GREEN, ORANGE; DISCREPANCY X3

199 Samoes green birefringence, apple-green birefringence Amyloid 2017; 24: 73-77. 1 **GREEN**; 1 GREEN, YELLOW; 2 YELLOW; DISCREPANCY X3, AGREE X1 [**2 NO GREEN**]

200 Samui green, yellow−green and golden-yellow birefringence AcsChemNeurosci 2019; 10: 2915-2918. 1 GREEN, YELLOW; 1 BLUE, YELLOW, ORANGE; 1 BLUE, YELLOW, RED, WHITE; DISCREPANCY X3 [**2 NO GREEN**]

201 Sandhu characteristic apple green, pathognomonic red green birefringence RetCases 2013; 7: 271-275. GREEN, ORANGE; DISCREPANCY X1

202 Santos green birefringence VetClinPath 2017; 46: 535-537. **GREEN**; AGREE X1

203 Sarkar typical apple green birefringence JOralMaxPath 2019; 23: 478. YELLOW, ORANGE, RED, WHITE; DISCREPANCY X1 [1 **NO GREEN**]

204 Sayed MISQUOTE pathognomonic green birefringence, apple green birefringence NDT 2014; 29: 2120-2126. [Puchtler H, Sweat F, Levine M. On the binding of Congo red by amyloid. J Histochem Cytochem 1962; 10: 355-364.] GREEN, YELLOW, WHITE; DISCREPANCY X1

205 Sayed diagnostic / pathognomonic green birefringence KidInt 2015; 87: 516-526. GREEN, YELLOW, WHITE;

ANCY X1

206 Scaglione MISQUOTE green birefringence; apple green birefringence ResVetSci 2013; 95: 569-571. [Bennhold H. Eine spezifische Amyloidfärbung mit Kongorot. Munch Med Woch 1922; 69: 1537-1538. Divry P, Florkin M. Sur les propriétés optiques de l’amyloïde. C R Sèances Soc Biol 1927; 97: 1808-1810.] YELLOW, GREEN; DISCREPANCY X1

207 Schmidt WRONG EXPRESSION red-green dichroism, green birefringence Amyloid 2019; 26: 118-124. GREEN, RED, WHITE, BLUE; DISCREPANCY X1

208 Schutz green birefringence, apple-green birefringence AngChemIntEd 2011 50: 5956-5960. 5 GREEN, YELLOW; DISCREPANCY X5

209 Scivetti classically apple-green, yellow, or orange birefringence; specific, typical green birefringence UltrastPath 2016; 40: 86-91. **GREEN**; AGREE X1

210 Scuderi green birefringence; apple-green birefringence SaudJOphth 2016; 30: 201-203. GREEN, YELLOW, WHITE; DISCREPANCY X1

211 Seethala apple-green birefringence IndPacElectJ 2010; 10: 3: 143-147. GREEN, YELLOW, ORANGE; DISCREPANCY X1

212 Sepulveda green birefringence NeurobiolAg 2014; 35: 472-481. GREEN, ORANGE; DISCREPANCY X1

213 Sergeeva apple-green birefringence Prion 2019; 13: 21-32. 2 GREEN, YELLOW; DISCREPANCY X2

214 Sethi apple-green birefringence AmJKidDis 2013; 61: 161-166. WHITE, ORANGE; DISCREPANCY X1 [1 **NO GREEN**]

215 Sethi characteristic apple-green birefringence KidInt 2012; 81: 201-206. GREEN, YELLOW, ORANGE, WHITE; DISCREPANCY X1

216 Sharma apple-green birefringence Cornea 2020; 39: 898-901. GREEN, YELLOW, RED; DISCREPANCY X1

217 Shidham characteristic apple-green birefringence JVisExp 2010; 2010/11/19 GREEN, YELLOW, RED; DISCREPANCY X1

218 Shientag green birefringence; characteristic apple-green birefringence CompMed 2016; 66: 225-234. **GREEN**; AGREE X1

219 Singh apple-green birefringence PNASUSA 2010; 107: 15069-15074. 4 YELLOW; DISCREPANCY X4 [**4 NO GREEN**]

220 Siniukova apple-green birefringence, yellow-green birefringence Prion 2020; 14: 278-282. 1 RED, WHITE; 1 GREEN, YELLOW; 1 POOR; DISCREPANCY X3 [**2 NO GREEN**]

221 Sirohi apple-green birefringence HumPath 2021; 93: 48-53. GREEN, YELLOW; DISCREPANCY X1

222 Sjolander ANOMALOUS apple-green birefringence; the anomalous color (green) as should be observed for proper Congo red birefringence Amyloid 2015; 22: 19-25. 2 **GREEN**; 3 GREEN, YELLOW; DISCREPANCY X3, AGREE X2

223 Snow positive red/ green birefringence SciRep 2019; 9: 561. WHITE, ORANGE; DISCREPANCY X1 [**1 NO GREEN**]

224 Sobue apple-green birefringence CardiovPath 2021; 51: 107315. GREEN, YELLOW, WHITE; DISCREPANCY X1

225 Son apple-green birefringence JKorMedSci 2014; 29: 145-148. YELLOW; DISCREPANCY X1 [1 **NO GREEN**]

226 Song apple-green birefringence RespMedCaseRep 2020; 31: 101223. YELLOW, RED; DISCREPANCY X1 [1 **NO GREEN**]

227 Sonthalia apple green birefringence WorldJHep 2016; 8: 340-344. 2 BLUE, YELLOW, ORANGE, WHITE; DISCREPANCY X2 [2 **NO GREEN**]

228 Sopova yellow-green birefringence SciRep 2019; 9: 18983. 3 GREEN, YELLOW; 1 POOR; 1 GREEN, YELLOW, ORANGE; DISCREPANCY X2, AGREE X3 [**1 NO GREEN**]

229 Stuhlmann-Laeisz typical green-yellow-orange birefringence VirchArch 2019; 474: 353-363. 2 GREEN, YELLOW; DISCREPANCY X2

230 Swaika typical green birefringence CaseRepGenet 2016; 9280812. ORANGE; DISCREPANCY X1 [**1 NO GREEN**]

231 Swanson apple-green birefringence JNeurolSurgB 2020; 81: 620-626. 1 BLUE, YELLOW, ORANGE, GREEN; 1 GREEN, YELLOW, ORANGE, WHITE; DISCREPANCY X2

232 Takahashi typical apple-green birefringence Amyloid 2014; 21: 211-215. **GREEN**; AGREE X1

233 Tamburro MISQUOTE apple-green birefringence, yellow-green Chiral 2010; 22: E56-66. [Divry P. Etude histochimique des plaques seniles. J Belge Neurol Psychiatr 1927; 27: 643-657.] 1 GREEN, WHITE; 1 RED, WHITE; DISCREPANCY X2 [**1 NO GREEN**]

234 Tambuzzi typical / classic apple-green birefringence JForensLegMed 2020; 71: 101939. POOR; DISCREPANCY X1 [1 **NO GREEN**]

235 Tan characteristic apple green birefringence DermatOnJ 2019; 25: 15. GREEN, YELLOW, ORANGE; DISCREPANCY X1

236 Tanskanen red to green birefringence NeuropathAppNeurob 2012; 38: 329-336. GREEN, YELLOW, WHITE; DISCREPANCY X1

237 Teng apple-green birefringence KidInt 2014; 86: 738-746. GREEN, YELLOW; DISCREPANCY X1

238 Terrier apple-green birefringence JMedCaseRep 2017; 11: 222. GREEN, YELLOW, RED; DISCREPANCY X1

239 Treibel typical apple green birefringence CircCardiovImag 2016; 9: 2016/08/12. 2 **GREEN**; AGREE X2

240 Tsai apple-green birefringence JOrthSurgRes 2017; 12: 194. 3 **GREEN**; AGREE X3

241 Tsiolaki Ham red–green birefringence, red-apple/green birefringence, green-apple birefringence FEBSLett 2015; 589: 159-164.

242 Tsiolaki Lou yellow to apple-green birefringence JStructBiol 2015; 191: 272-280. 1 GREEN, YELLOW, ORANGE; 1 YELLOW, ORANGE; DISCREPANCY X2 [**1 NO GREEN**]

243 Tsuji yellow-green birefringence BMCSurg 2016; 16: 62. GREEN, YELLOW; AGREE X1

244 Tsukawaki emerald-green birefringence; green birefringence BiomedMater 2016; 11: 065010. 3 **GREEN**; AGREE X3

245 Uchihara apple-green birefringence PathResPract 2018; 214: 1661-1666. 3 **GREEN**; AGREE X3

246 Usnarska-Zubkiewicz green AdvClinExpMed 2014; 23: 235-244. **GREEN**; AGREE X1

247 van Gameren characteristic apple-green birefringence; green birefringence ArthCareRes 2010; 62: 296-301. 4 **GREEN**; AGREE X4

248 Venkatesh apple green birefringence Ophthalm 2017; 124: 1014-1022. RED; DISCREPANCY X1 [1 **NO GREEN**]

249 Visconte typical green birefringence JThrombHem 2020; 18: 3029-3042. 2 GREEN, YELLOW; DISCREPANCY X2

250 Wenson apple-green birefringence JCutPath 2012; 39: 263-269. WHITE; DISCREPANCY X1 [1 **NO GREEN**]

251 Wetwittayakhlang typical apple-green birefringence CaseRepGastr 2019; 13: 462-467. GREEN, YELLOW; DISCREPANCY X1

252 Woldemeskel green birefringence, yellow-green birefringence VetMedInt 2012: 427296. **GREEN**; AGREE X1

253 Wong green birefringence PNASUSA 2010; 107: 1977-1982. **GREEN**; AGREE X1

254 Yakupova MISQUOTE and ANOMALOUS apple-green birefringence, anomalous colours (yellow/green and blue/green), yellow to apple-green birefringence BiosciRep 2019; 39: 2018/12/21. [Howie AJ, Owen-Casey MP. Discrepancies between descriptions and illustrations of colours in Congo red-stained amyloid, and explanation of discrepant colours. Amyloid 2010; 17: 109-117.] BLUE, GREEN, YELLOW, ORANGE, RED; DISCREPANCY X1

255 Yakupova MISQUOTE yellow to apple-green birefringence, yellow/green birefringence JBiomolStructDyn 2018; 36: 2237-2248. [Howie AJ, Owen-Casey MP. Discrepancies between descriptions and illustrations of colours in Congo red-stained amyloid, and explanation of discrepant colours. Amyloid 2010; 17: 109-117.]

256 Yamada apple-green birefringence ErjOpenRes 2020; 6: 3. **GREEN**; AGREE X1

257 Zussy green birefringence AmJPath 2011; 179: 315-334. 1 GREEN, ORANGE; 2 GREEN, YELLOW, ORANGE; DISCREPANCY X3
